# ‘To use or not to use’: a qualitative study to evaluate experiences of healthcare providers and patients with the assessment of burden of COPD (ABC) tool

**DOI:** 10.1038/npjpcrm.2016.74

**Published:** 2016-11-17

**Authors:** Annerika H M Slok, Mascha Twellaar, Leslie Jutbo, Daniel Kotz, Niels H Chavannes, Sebastiaan Holverda, Philippe L Salomé, P N Richard Dekhuijzen, Maureen P M H Rutten-van Mölken, Denise Schuiten, Johannes C C M in ’t Veen, Onno C P van Schayck

**Affiliations:** 1 CAPHRI School for Public Health and Primary Care, Department of Family medicine, Maastricht University, Maastricht, The Netherlands; 2 Institute of General Practice, Medical Faculty of The Heinrich-Heine-University Düsseldorf, Düsseldorf, Germany; 3 Leiden University Medical Centre, Department of Public Health and Primary Care, Leiden, The Netherlands; 4 Lung Foundation Netherlands, Amersfoort, The Netherlands; 5 Huisartsencoöperatie PreventZorg, Bilthoven, The Netherlands; 6 Department of Pulmonary Diseases, Radboud University medical centre, Nijmegen, The Netherlands; 7 Erasmus University Rotterdam, Institute for Health Policy and Management/Institute for Medical Technology Assessment, Rotterdam, The Netherlands; 8 PICASSO Care Optimisation Program, Alkmaar, The Netherlands; 9 Sint Franciscus Vlietland Groep, Department of Pulmonology and SZT Centre of Excellence for Asthma & COPD, Rotterdam, The Netherlands

## Abstract

In the management of chronic conditions, such as chronic obstructive pulmonary disease (COPD), there is a shift from doctor-driven care to patient-centred integrated care with active involvement of and self-management by the patient. A recently developed tool, the assessment of burden of COPD (ABC) tool, can be used in this transition to facilitate self-management support and shared decision-making. We performed a qualitative study, in which we collected and analysed the data using the methods of conventional content analyses. We performed in-depth interviews consisting of mainly open questions. Fifteen healthcare providers and 21 patients were interviewed who had worked with the ABC tool in daily care. In general, participants responded positively to the tool. Healthcare providers felt the visual representation provided was effective and comprehensible for patients and provided them with insight into their disease, a finding that patients confirmed. If patients were allowed to choose between a consultation with or without the ABC tool, the majority would prefer using the tool: it provides them with an overview and insight, which makes it easier to discuss all relevant topics related to COPD. The tool can provide structure in consultations, and is compatible with the concepts of ‘motivational interviewing’ and ‘individualised care-planning’. Suggestions for improvement related to content and layout. So far, the tool has only been available as a stand-alone online program, that is not connected to the electronic medical record systems. It was therefore suggested that the tool be integrated into the systems to enhance its usability and its uptake by healthcare providers.

## Introduction

In chronic conditions such as chronic obstructive pulmonary disease (COPD), there is a shift from doctor-driven care towards more patient-centred integrated care with active involvement of and self-management by the patient.^1,2^ This requires patients to take a more active role in managing their own disease.^3,4^ Self-management^5^ and shared decision-making^6–8^ appear to be essential to learning to cope with the disease,^9^ improving health status,^10,11^ reducing the use of care services and hospital admissions.^10–12^ This process requires a behavioural change on the part of both healthcare providers and patients.

In Western countries, healthcare providers are becoming increasingly better trained in this transition, for example by acquiring the skills of motivational interviewing,^13^ and integrating the different components of shared decision-making into their consultations.^14^ Despite this training, healthcare providers and patients are often not yet used to this approach.^15^ Patients need to be properly educated in order to manage their disease effectively,^9^ although knowledge alone is not enough to change behaviour.^16^ They should also be encouraged and trained to take control of their illness, its treatment and consequences.^2^ In addition, patients need to receive support in their new and active role in shared decision-making.

**Figure 1:**
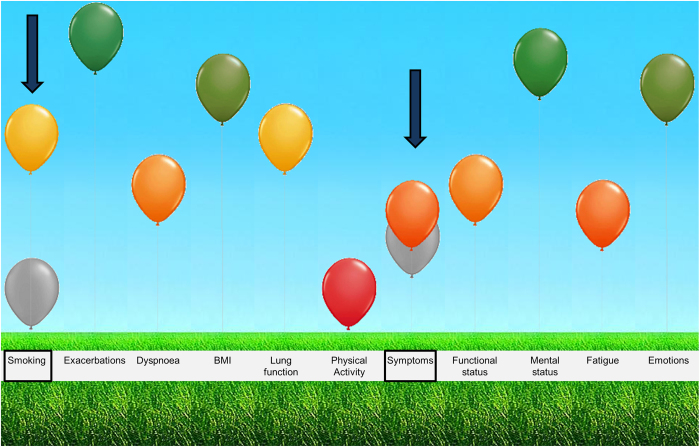
Overview of the integrated health status of a COPD patient, showing a change in smoking behaviour and symptoms since the previous visit, as indicated by the arrows. A change is visualised by adding the scores of previous assessment in the display with grey balloons.

This process, which is a joint venture between healthcare provider and patient, is likely to be facilitated by the recently developed assessment of burden of COPD (ABC) tool. The ABC tool is currently developed as a stand-alone online computer program that healthcare providers can access using login codes they received from the researchers prior to the study. The ABC tool starts with a patients’ reported burden of COPD, using the ABC scale. By adding other parameters to the ABC scale, such as smoking status and lung function parameters, the integrated health status of a COPD patient is assessed. The ABC tool then visualises this integrated health status by means of a balloon diagram (Figure 1).^17^ The scores of the current assessment are shown with the coloured balloons, and the scores of the previous visit are shown with grey balloons, which aids in monitoring any changes in the patients’ status. The balloon diagram facilitates communication between the healthcare provider and patient on health-related topics that affect the patient’s COPD burden.

We hypothesise that integration and visualisation of the different aspects of COPD helps healthcare providers, and patients develop an awareness of the different components associated with the experienced burden. If patients experience more symptoms, for example, they can easily relate this to their smoking behaviour (Figure 1), which then makes them more motivated to quit.^18,19^ The ABC tool also uses a treatment algorithm with treatment advice, based on current COPD healthcare guidelines. The visual display could be used in shared decision-making between patients and healthcare providers, by integrating the coherence of the domains, e.g., symptoms and smoking status, supporting a personalised treatment plan with behavioural change. As the majority of COPD treatment options relate to behavioural change management, it stresses the importance of patients managing their own disease and treatment plan.^5^

As the ABC tool is a newly developed tool, it is important to evaluate its performance. The psychometric properties of the scale and the effectiveness of the tool on different outcome measures have already been investigated in a cluster randomised controlled trial (RCT). The ABC scale was found to be a valid and reliable questionnaire. Moreover, results of the RCT showed that when the ABC tool is used, patients report a better disease-specific quality of life and a better perceived quality of care. These results are reported elsewhere.^20,21^ In addition, it is important to conduct an in-depth investigation of the experiences of patients and healthcare providers who have already used the tool in daily care, in order to discover whether users of the ABC tool consider it a valuable contribution to the usual care.

Therefore, the aim of this study was to conduct a qualitative evaluation to assess healthcare providers’ and patients’ experiences with the ABC tool, by examining (1) the opinion of the healthcare providers and patients about the different components of the ABC tool; (2) the extent to which the ABC tool is useful in daily COPD care; and (3) the factors that would facilitate and hamper possible future implementation.

## Results

We performed 15 individual interviews with healthcare providers. The interviews lasted between 23 and 55 min. Characteristics of the healthcare providers are shown in Table 1. We also performed 21 individual interviews with COPD patients who participated in the RCT, who were treated by eleven different healthcare providers from five hospitals and six primary care practices. The interviews lasted ~35 min. Characteristics of the patients are shown in Table 2.

### Content and components of the ABC tool

#### ABC scale

According to healthcare providers, the fact that the ABC tool is based on the clinical COPD questionnaire is a significant strength. The COPD questionnaire is a commonly used questionnaire in the Netherlands and has been proven to be simple and quick, but valid, measuring the clinical status of the airways, functional limitations and psychosocial dysfunction.^22–24^ The healthcare providers mentioned that the four additional items, three questions measuring emotions and one question measuring fatigue, were useful and provided greater insight into the burden of COPD experienced by the patient.

Patients also appreciated the questions assessing emotions as these influenced their perception of the disease burden. Assessing and visualising emotions helped them discuss this difficult topic. On the other hand, some did not understand why emotions were covered in the questionnaire, as they did not believe that emotions were directly related to COPD. However, the questions did not bother these patients, either, and they were willing to answer them.

*‘Emotions and feelings…. They are very important. (….) They have a major impact. And of course your physical health and dyspnoea. But also those emotions. Fatigue and emotions are also related.’*—Patient 4

We asked healthcare providers and patients where the ABC scale was administered, as it was designed to be self-administered. The majority completed the questionnaire in the waiting room, though several did so in the consultation room and others sometimes in the waiting room and sometimes in the consultations room.

Regarding completion time, patients mentioned that filling out the questionnaire took them only a few minutes and that it required minimal effort to do so prior to each consultation. Although they did not report having trouble understanding the language in the questionnaire, some felt that seven response options might be too many, as it was difficult to distinguish between ‘a few times’ and ‘several times’, for example.

Furthermore, some patients mentioned that it is sometimes difficult to answer the questions because of the day-to-day variations of the disease, and because they suffer from other conditions as well, which makes it difficult to know whether the complaints are related to their COPD or other diseases, such as diabetes.

#### Visual representation of integrated health status

The healthcare providers considered the visual representation, both conceptually and as regards the illustration with coloured balloons, to be a key strength of the ABC tool. They thought it was understandable for patients, and felt that it provided the patient greater insight into the various aspects of COPD and how they interrelate. Healthcare providers also welcomed the fact that the program provides an immediate overview after the results of all items are entered into the computer, which they considered helpful both for themselves and for the patients.

*‘Patients can watch it on the computer screen. They can understand everything and it is also easy to grasp.’*—PN 2

*‘You immediately have an overview, so you do not have to explicitly ask all these questions, because you already have them in your overview.’*—PCNS 2

*‘And what I think is fantastic, is the balloon system. Actually, for me that is the most valuable part.’*—PCNS 1

All patients mentioned that it was clear that the positions of the balloons in the diagram were determined by their answers to the questions. They were generally positive about the visual representation. Their first impressions varied: most patients liked it and said it was fun and interesting, whereas others found it was a bit childish or strange. Most patients felt that the visual display clarified the integrated health status and that balloons were better-suited to visualising burden of COPD, than other options such as smileys, traffic-lights or graphs. The added value of the visual display over words was emphasised, as most patients were able to remember more of what the healthcare providers told them when the balloons were used as an aid. Furthermore, most patients found it stimulating and motivating to see the different balloons per domain. Not all patients necessarily responded positively: some patients had a neutral reaction and no real opinion or expectations. Some patients felt it was unnecessary to use a visual display and a few preferred graphs over an image with balloons.

*I think that nine out of ten patients feel that this is better than when it is only discussed or when you have to read it. (…) This provides immediate insight. If I were this patient (interviewer brought a visual display of a test patient), then I would think: ‘I have to do something with this. What can we do?’*—Patient 6

*‘Especially with the colours that they can see… this is good, and this is not so good. I have to work on this, I don’t have to worry about that. Those are surely plusses. And it’s a good tool for less-educated patients.’* Patient 2

#### Grey balloons for monitoring

The grey balloons, which showed a comparison with the scores of the previous consultation, were considered a useful addition by the healthcare providers, as they immediately showed the patients’ clinical stability, improvement or deterioration. Patients tended to forget their previous health status once 6 months had passed between the two medical check-ups, so showing previous health status as well as current health status is considered to be a good reminder.

Some patients did not remember having seen the grey balloons. However, when the purpose of the grey balloons was explained by the interviewer, they did understand it and thought it was very clear. Most of them felt that including grey balloons to monitor progression and deterioration stimulated and motivated them to take action. They thought that having this information would encourage conversation with their healthcare provider about what caused the changes in their experienced burden and possible treatment options.

Patient: *‘I thought it was important, because I also started working quite intensively on my physical fitness, and the balloons indeed show the results.’ Interviewer: ‘And how do you feel about the visualisation of these results?’ Patient: ‘Well, it motivates you.’*—Patient 9

### Usability and users

Several healthcare providers thought that the ABC tool could have an important role in the transfer of patients between different sectors of care, for instance between primary and hospital care, and between pulmonologists and PCNSs. The tool could thus be used as standard for all healthcare providers involved in patient care and might improve continuity of the care.

It took some healthcare providers a while to get acquainted with the tool. Nevertheless, after figuring out the program they eventually found it easy to access the tool and add a new patient and felt the composition was logical.

*‘Yes, it speaks for itself, it is very simple.’*—PN 1

Several healthcare providers and patients thought that mainly PNs and PCNSs would start using the program. They usually have more time per patient than pulmonologists and GPs had, and have therefore more time to use the tool as the researchers intended, namely to increase patient involvement in the treatment of their disease and promote self-management.

*‘The tool would be mainly usable for the practice nurses, if I am truly honest, I think they are more consistent than we are. They also have more time.’*—GP1

The majority of the healthcare providers indicated that the ABC tool was useful for the entire COPD patient population, and especially for new patients. Patients confirmed this finding, and added that the visual display might be especially helpful for older or less-educated patients or for patients with lower health literacy. The tool might not be suitable for patients who have difficulties filling out the ABC scale, such as those who are not fully able to understand the Dutch language because of, for example, low literacy or Dutch not being their native language.

*‘Well I would use it for every COPD patient.’*—PN 4

### Purpose and added value of the ABC tool

Patients were asked whether they understood the purpose of the ABC tool, and why it was developed. Patients who understood the question indicated that it was developed to show how they are doing, to monitor improvement and deterioration, and in which domains there was room for improvement. Some patients indicated that the purpose was to highlight changes in their health and their condition and in domains that have room for improvement. Not all patients understood the question from the researcher about the purpose of the ABC tool initially, but they did after it was explained.

*‘You want to visualise it and I think you want to visualise what has gone well and what needs working on.’*—Patient 19

#### Complete overview and structure

Most healthcare providers and patients felt that the ABC tool was complete and representative for COPD. It was stated by both healthcare providers and patients that during consultations, the ABC tool provides structure, and it looks at aspects which are all relevant to COPD. Several strengths of the tool were mentioned: as all relevant topics are visualised, nothing is left out; it aids in the discussion of difficult topics, such as psychosocial aspects and smoking cessation; it provides insight into the disease and the possible treatment options; and it makes patients aware of the things they can do something about themselves. Some patients did not share this opinion, but mentioned the added value of being able to monitor progression or deterioration.

*‘You can provide insight, and you can make sure that you do not forget the most important aspects (…) you create the opportunity to work according to a somewhat structured format, a step-by-step plan’*—Pulmonologist 1

*‘(…) because it highlight the problems…it is easier to get to the heart of the matter.’*—PCNS 3

*‘Because of the balloons I have become more aware of things. More aware of things I can do something about, what else I can do myself to improve it, to stay in better shape.’*—Patient 10

*‘I feel like everything: smoking, exacerbations, dyspnoea, everything will be discussed because of the balloons. And without the balloons, we randomly discuss things that I mention. And now you really discuss all balloons.’*—Patient 12

#### Personalised care and patient involvement

Healthcare providers mentioned that it could be helpful for the less experienced healthcare providers and might encourage the more experienced providers to think outside their routine. The program provided action points for shared decision-making with the patient, which also led to more meaningful discussions in the consultation.

The tool fitted in well with the concept of ‘motivational interviewing’ as well as ‘individualised care planning’ that are currently relevant topics for healthcare providers. It helped healthcare providers discuss behavioural change with patients. Furthermore, healthcare providers felt that it forced the patients to think about their personal goals and a treatment plan. Especially if consultations were frequent, healthcare providers tended to notice an increase in their patients' proactivity with regard to the treatment plan.

Some patients mentioned the importance of setting personal goals, especially if their health deteriorated. By contrast, some patients did not feel this was necessary, and had no expectations of the consultations with their healthcare providers in terms of treatment plans. They preferred to continue behaving as they had in the past without setting new goals. When questioned, most patients confirmed the importance of being involved in the decision-making process of their treatment plan. A few patients preferred that the treatment plan be determined by the healthcare provider.

Interviewer: *‘Have you ever discussed with your pulmonary nurse what you would like to achieve? Whether you have a goal?’ Patient: ‘No, never. I will see what happens. We cannot discuss this because I cannot predict the future.’*—Patient 7

After each consultation, an overview of the balloons and the treatment plan could be printed. We asked patients whether they had received the print out from their healthcare provider and whether they did anything with this overview. A few patients mentioned that they had discussed it with their partner, but most patients reported that they did not do anything with the overview and had thrown it away.

### Suggestions for improvement and implementation

#### Layout and content

Some improvements were suggested concerning the layout of the program, such as the addition of a ‘home-button’. Some suggestions were also made regarding the content of the ABC tool. It was recommended that personal goals from previous consultations would be shown in order to refer to and reflect on these goals with the patient. This could facilitate a conversation about how the patient is progressing towards achieving these goals. In addition, they felt it would be necessary to qualify the disease burden as mild, moderate or severe, as the health insurance companies would require this.

#### Treatment algorithm

Some healthcare providers missed a few items in the treatment algorithm, such as inhalation instructions for patients, vital signs (pulse rate and blood pressure) and adaptation or coping. Furthermore, several of them believed that some of the treatment options were too general and not sufficiently concrete, especially those that advised referral of the patient to other healthcare providers. One of the healthcare providers stated that there was a risk that the tool would at one point provide insufficient opportunity to deviate from protocols if they believed doing so would benefit their patient. Some healthcare providers suggested that the treatment advice should be formulated such that it is easier for patients to understand and that obscure abbreviations such as ‘ICS’ (inhalation corticosteroid) should be avoided.

*‘Yes, the treatment options are very general. They are not so specific that you think…okay…this is useful for patients.’*—PN 3

#### Implementation of the tool in COPD care

Almost all healthcare providers agreed that the ABC tool must be implemented in the electronic medical record (EMR) systems of the GP practice or hospital. As the ABC tool had not yet been integrated into the EMR systems by the time the RCT took place, using the tool was time-consuming, because of the extra preparation required and the fact that all the data had to be transferred from the ABC tool into the EMR systems.

*‘It takes longer, because you have to transfer data and information.’*—PN 1

Therefore, this information should preferably be transferred automatically from one system to another. No specific preference was expressed as to whether the ABC tool should be fully integrated into or be linked to the EMR systems. Even if the tool could not be integrated, several healthcare providers would still use it, but only for a select group of COPD patients, although some would not, as it would be too labour-intensive because of the extra preparations and entry of data in two systems.

*‘It should really be integrated into the electronic medical record systems.’*—PN 3

*‘I can do this with the 10 patients from the RCT…but I cannot…. I cannot work with two systems if I have a busy schedule.’*—PCNS 1

It was suggested that a concise manual be developed that provides practical instructions on how to use the program efficiently in a consultation, explaining for example what preparations should be made before seeing a patient and how to start a conversation with them. In addition, it would be helpful to create a ‘dummy’ patient or an instructional video to learn about the program before using it in a consultation with a real patient. The development of a workshop or training was also proposed. These would need to focus on how to start a conversation with a COPD patient and complete it efficiently, as well as how to optimally use the ABC tool for shared decision-making and individualised care planning.

*‘If the ABC tool is going to be implemented, I think it will be very important that a good manual is made available.’*—PCNS 3

*‘There should be a ‘dummy’ patient. They could fill out the ABC tool and you could see what happens.’*—PN 1

*‘If you had made an instructional video, I would have watched it.’*—PCNS 4

The interviews with patients were concluded with the question: ‘Would you prefer to use the ABC tool with the balloons in the next consultation with your healthcare provider, or not?’ The majority of the patients would prefer using the ABC tool, because they thought that the consultation was much clearer when the ABC tool was used and because it provides a good overview of their changes that have occurred since previous consultations. Only a few patients felt it was unnecessary, but they said they would not mind if the ABC tool were used.

*‘Well… you can see that for yourself how it....or if there has indeed been a change. If something changed in my conversations, in my dyspnoea, in everything. So, I would choose to have the balloons.’*—Patient 21

## Discussion

### Main findings

The ABC tool has been developed to guide patients and healthcare providers towards making a tailor-made self-management plan. The tool can facilitate a structured consultation and can provide insight into the patients’ disease and different treatment options. The healthcare providers and patients who participated in the interviews were positive about this newly developed ABC tool. Healthcare providers thought the ABC tool’s visual representation was effective and comprehensible for patients and provided them insight into their disease, a finding that was confirmed by patients. The tool not only provides structure for the consultations, but it is also compatible with the concepts of ‘motivational interviewing’ (e.g., increasing patients’ motivation to change behaviour, by for example focusing on a balloon that has room for improvement), ‘individualised care planning’ and it supports shared decision-making. The tool was considered to be user-friendly and was easy to use in the consultation. It is likely to be used mainly by PNs and PCNSs, as they have more time per patient.

In its current form, the daily use was hindered by the extra preparation required (for instance, logging into the program and adding new patients) and the need to record patient information in both the ABC tool and the EMR system. Therefore, it was suggested that the tool be integrated into the EMR systems’ healthcare providers already use. Implementation of the tool in EMR systems is feasible but challenging, as there are many EMR systems for hospitals and primary care practices in the Netherlands. Possible options for implementation could be integrating the ABC tool in the different EMR systems or making a central application developed by one central party that maintains a linkage between the ABC tool to the different EMR systems.

A striking finding was the lack of recollection of the grey balloons by most patients. They were added to the programme to show previous health status, in order to monitor disease progression. This finding might be partly attributed to recall bias and the fact that the duration of the study was only 18 months, which might have been too short a time to become familiar with the tool. It could also be explained by the fact that most patients remained stable, so no grey balloons were actually shown. Furthermore, various studies investigated the effects of colours on emotions and memory, and results indicate a relation.^25–29^ Therefore, further research could be conducted to investigate patients’ preferences regarding the colours and the effects of the coloured balloons on the patients’ emotions and memory in the context of the ABC tool.

### Interpretation of findings in relation to previously published work

The use of a tool like the ABC tool is in line with the latest insights regarding the support of patients with chronic conditions in self-management action planning.^30,31^ Nowadays, healthcare providers seem to be more familiar with these concepts, but still tend to focus on biomedical outcomes,^15,32^ also indicated in the current study by some healthcare providers who mentioned that certain topics, such as emotions, are difficult to discuss with patients. Moreover, patients seem to be unfamiliar with the term ‘care-planning’^33^ and tend to arrive at consultations without any expectations and without being prepared, displaying a passive attitude,^15^ which was also found in our study. Patients were asked about their expectations of the regular check-ups and their personal goals, but some replied that they had no expectations at all, that they would just come to a consultation because their healthcare provider invited them and that they had no specific goals, as they knew that getting better was not possible. This indicates that patients should be encouraged and supported to change their approach and have a more active role in consultations. Patients need support in order to be able do this, and the ABC tool is an easy facilitator in this respect. When deciding on a treatment plan, patients and healthcare providers should work together and form an alliance,^34^ to promote better communication^35^ and greater medication adherence.^36,37^

Not only will patients have to change their behaviour, but so will the healthcare providers,^34^ also indicated by some of the healthcare providers in this study, who suggested that the tool could be helpful for some healthcare providers to think outside their routine. The ABC tool can be used to facilitate self-management action planning, by first reporting experienced burden of COPD and deciding on a self-management domain by discussing the domains with an orange or red balloon or balloon showing deterioration.^34^

### Strengths and limitations of this study

A strength of this study was the heterogeneity in the characteristics of the respondents. We conducted interviews with healthcare providers and patients from both primary and hospital care, and within the healthcare provider group, we interviewed physicians as well as practice nurses and nurse specialists. This covers the intended population of healthcare providers and patients who will be using the ABC tool if it is implemented. The fact that some healthcare providers participating in the RCT were already more engaged in COPD care and, consequently, tended to be enthusiastic about the ABC tool from the start is one possible limitation of the study. In contrast, these healthcare providers could have been more critical due to higher expectations about the developed tool. Furthermore, not every patient could remember that the ABC tool was used in the consultation, despite the fact that the completion of the ABC scale was registered at least twice for each patient, as shown in Table 2. Therefore, sometimes the answers might have been based on hypothetical situations, instead of being real memories. In addition, although the researcher repeatedly explained that there were no right or wrong answers, patients may have given socially desirable answers.^38^

### Implications for future research, policy and practice

In order for the ABC tool to be used efficiently in consultations, it must be integrated into the EMR systems. In addition, some revisions to the tool might render it more user-friendly, such as adding a ‘home-button’ and showing the personalised treatment plan of previous consultation. Furthermore, the treatment options have to be updated according to current guidelines, and adjusted as suggested during the interviews. Healthcare providers should be instructed on how to use the tool effectively during consultations, to promote self-management and guide patients towards goal-setting. For this purpose, an instructional video and a workshop would be useful.

It is important that healthcare provider empower patients, so that patients arrive at consultations prepared and aware of their current health status, switching from being a passive recipient to an active participant.^39^ Taking on an active role in creating and applying an appropriate self-management plan has an impact on the ability of coping with the disease, health status, use of care services and hospital admissions.^10–12^ Further research should focus on implementation and on how the ABC tool can be used to help patients create a personalised treatment plan and strengthen their self-management skills.

### Conclusions

The ABC tool appears to be a very useful instrument for consultations with COPD patients. Both patients and healthcare providers believe the tool offers added value and therefore recommend that the ABC tool should be integrated into regular care and consequently into the EMR systems of healthcare providers.

## Materials and methods

### Design

A qualitative study was performed, and as our aim was to describe the reactions and experiences of ABC tool users, the methods of conventional content analysis were used.^40^ Individual interviews were performed to collect information. This study was approved by the Medical Ethics committee of Atrium-Orbis-Zuyd hospital, The Netherlands.

### Information collection

The data were collected from interviews consisting of mainly open-ended questions. Topic lists were used in the individual interviews. These are included in Supplementary Appendix 1 and 2. Topics that were relevant for answering the research questions were decided upon by different researchers involved in this project, who reached consensus by discussion.

The interviews with healthcare providers were conducted by LJ, under supervision of AHMS, at the practices of the healthcare providers. Patients were interviewed by AHMS, at home or in the hospitals, depending on their preference and practical or logistical reasons. AHMS had previous experience in interviewing patients for research purposes, and L.J. was extensively trained in interviewing during her medical education. Consistency between interviews was warranted by AHMS being present during all interviews.

### Study population

We approached 30 healthcare providers (general practitioners (GPs), pulmonologists, practice nurses (PNs) and pulmonary clinical nurse specialists (PCNS), who were previously randomly allocated to the intervention group of the RCT by e-mail and subsequently by telephone to investigate their willingness to participate in an individual interview. In total, 28 persons indicated that they were willing to participate in the interviews. One PN and one PCNS declined participation because of a busy work schedule.

We selected a total of 15 providers (2 GPs, one pulmonologist, 5 PN, 6 PCNSs and 1 healthcare provider who works both as PN and PCNS) for a face-to-face interview to discuss how the ABC tool was used in both primary and hospital care. Participants were mainly PNs and PCNSs, as they have a central role in the use of the ABC tool. Our selection criteria were healthcare providers from both primary and hospital care and from different places in the Netherlands to ensure a good geographical spread, in order to prevent potential cultural differences. After performing nine interviews, we observed data saturation.

We approached 160 patients who were randomised to the intervention group of the RCT to participate in an individual interview by adding a reply card to the final questionnaire of the RCT.^41^ This reply card included postage for mailing, the address of the university and check boxes to indicate whether the researchers were allowed to contact the patient by telephone or not. Over 60 patients (almost 40%) replied that they were willing to be contacted for an interview. When planning the interviews, we intentionally considered patients from both genders and from both primary care and hospital care. Patients were contacted by the researcher, and if they decided to participate an appointment was made. Patient information and an informed consent form were sent to the patient by mail, who was asked to sign the consent form and give them to the researcher prior to the interview. After performing 15 interviews, we observed data saturation. We interviewed another 6 patients, bringing the patient total to 21, thereby achieving a balance between primary and hospital care.

### Analysis

All interviews were audio-recorded and transcribed verbatim. Data were analysed according to the conventional content analysis, using specialised software called QSR Nvivo9.^42^ Data collection and analysis did not occur simultaneously. In order to familiarise ourselves with the data, we started by reading all transcripts in detail. We then highlighted phrases that captured the key concepts. Codes were derived from the data during the data analysis (inductive coding).^40^ These initial steps were performed independently by two different individuals: the healthcare provider interviews by LJ and AHMS and the patient interviews by MT and AHMS. The researchers reached consensus on coding through discussion. Codes were then related, linked and divided into categories.^40^

## Figures and Tables

**Table 1:** Characteristics of the healthcare providers

*Interviewed healthcare providers*	n	*Gender, (M/F) (*n*)*	*Age, range (years)*	*Experience in (COPD) healthcare, range (years)*
GP	2	2/0	52–61	23–42
Pulmonologists	1	1/0	52	25
PN	5	0/5	44–53	12–30
PCNS	6	1/6	29–52	8–32
Nurse practitioner and nurse specialist	1	0/1	52	29

Abbreviations: COPD, chronic obstructive pulmonary disease; F, female; GP, general practitioners; PCNS, pulmonary clinical nurse specialists; PN, practice nurses; M, male.

**Table 2:** Characteristics of the patients

	*Gender (M/F)*	*Age*	*Healthcare setting*	*Number of times ABC tool used*^a^
1	F	67	Hospital care	2
2	M	56	Hospital care	4
3	F	72	Hospital care	4
4	F	69	Hospital care	3
5	M	64	Hospital care	4
6	M	72	Hospital care	4
7	F	70	Primary care	3
8	M	79	Primary care	4
9	M	65	Hospital care	3
10	F	57	Hospital care	4
11	M	70	Hospital care	4
12	F	52	Hospital care	4
13	M	67	Hospital care	4
14	M	66	Hospital care	4
15	F	69	Hospital care	3
16	F	53	Primary care	4
17	F	59	Primary care	4
18	M	63	Primary care	4
19	M	73	Primary care	4
20	F	72	Primary care	4
21	F	71	Primary care	4

Abbreviations: ABC, assessment of burden of COPD; COPD, chronic obstructive pilmoary disease; F, female; M, male.

a Number of times the ABC scale was completed during consultation according to the registration system of the cluster randomised controlled trial.
