# Supplementary material for: ‘To use or not to use’: a qualitative study to evaluate experiences of healthcare providers and patients with the assessment of burden of COPD (ABC) tool
Source: NPJ Prim Care Respir Med. 2016 Nov 17;26:16074–. doi: 10.1038/npjpcrm.2016.74 (PMC5113148; doi:10.1038/npjpcrm.2016.74)
Supplement: Supplementary Appendix 1 [file npjpcrm201674-s1.doc]

**APPENDIX 1**

**Interview guide for individual interviews with healthcare providers (originally in Dutch)**

1. What is your opinion on the user-friendliness of the program?
2. What is your opinion on the lay-out?
   1. Do you think that the visual concept of the balloons is clear for patients?
3. Currently patients fill out the ABC scale in the waiting room, how does this work?
   1. What is your opinion on this?
   2. If this does not function well, do you have any suggestions for improvements?
4. On what basis did you include your patients in the trial?
   1. What reasons did you have for not including patients?
5. What do you consider as strengths of the program?
6. What do you consider as limitations of the program?
7. Could the program be used in its current form? If no:
   1. What are points of improvement with regard to user-friendliness?
   2. What are points of improvement with regard to the content of the algorithm?
8. Does the program cover all the relevant aspects in the treatment of COPD or are there factors that are overlooked?
9. How does the consultation proceed with patients while using the program?
   1. Does the program add certain features to your usual level of care with regard to COPD patients?
   2. Do you approach things differently than you normally do?
      1. If yes, could you give us an example?
      2. Does this in your opinion add value to the treatment of COPD?
   3. Does the program make you discuss other things with patients than normal?
      1. If yes, could you give us an example?
      2. Do you acquire different information than normal? Do you consider this relevant and of added value?
10. Do you think that patients have a different way of coping with COPD because of the program?
    1. Does it encourage patients to actively contribute to the treatment plan and to subsequently stick to it?
       1. Are patients able to formulate a personal objective?
    2. Does the treatment plan match the patients’ needs better?
11. Would you recommend the program to colleagues ?
12. Suppose the program would ultimately not be implemented into the information systems, would you still use it?
    1. If yes, could you briefly explain why?
    2. If no, could you briefly explain why not?
13. Which factors could hinder the implementation of the program in your practice or department?
14. If we were to organize a workshop about the ABC tool and computer program in the future, what should we focus on?
