# Supplementary material for: ‘To use or not to use’: a qualitative study to evaluate experiences of healthcare providers and patients with the assessment of burden of COPD (ABC) tool
Source: NPJ Prim Care Respir Med. 2016 Nov 17;26:16074–. doi: 10.1038/npjpcrm.2016.74 (PMC5113148; doi:10.1038/npjpcrm.2016.74)
Supplement: Supplementary Appendix 2 [file npjpcrm201674-s2.doc]

**APPENDIX 2**

**Interview guide for individual interviews with patients (originally in Dutch)**

1. Can you remember your last consultation with your healthcare provider? What happened during this consultation?

*“Researcher shows the visual display with balloons”*

1. Can you remember these balloons?
2. Can you tell me something about these balloons?
3. Which topics did you discuss compared to normal?
4. What do you think is the purpose of these balloons?
5. How do you feel about active participation during consultation?
6. Which personal goal did you formulate during last consultation?
7. What do you do at home with what you have heard during consultation? Is this changed compared to usual?
8. What do you consider as strengths of the program?
9. What do you consider as limitations of the program?

Additional questions if necessary:

1. How did you experience the provided healthcare in the past 1.5 year?
2. Was there a difference in comparison with before using the balloons?
3. How do you feel about completing a questionnaire?
4. How do you feel about the questions?
5. How long did it take to complete the questionnaire?
6. Did you have enough time to complete the questionnaire?
7. Where would you like to complete the questionnaire (at home, waiting room/consultation room)?
8. How do you feel about the balloons?
9. Do you think the balloon-picture gives a complete overview of your COPD or health?
10. Do you think the picture is clear?
11. How do you feel about formulating a personal goal?
12. How do you feel about using the balloons during every consultation?
13. How do you feel about monitoring your progression or deterioration?
14. Did you have an overview of your treatment plan at the end of the consultation?
15. Do you discuss things now with your healthcare provider that you did not discuss before (the ABC tool was used)?
16. Would you recommend the tool to other COPD patients?
17. Would you like to use the tool with the balloons during next consultation?
